# Dr. Van Emen—Bellows and Blow Pipe

**Published:** 1849-07

**Authors:** 


### DR. VAN EMEN—BELLOWS AND BLOW PIPE.

Is composed of three circular boards, nine inches in diameter, and one-half in thickness, made of one-fourth inch stuff, glued together cross wise. The middle one is joined to the lower at one edge, and to the upper at the opposite edge, by a back flap hinge. Through the lower and middle boards, are made air holes, one-fourth of an inch in diameter; over these is placed a piece of india rubber cloth, or soft, close leather, two inches wide, and two and one-half long, and tacked across the ends, the sides left free. Between the lower and middle boards, is placed one half of a strong wire sofa spring.

A strap of india rubber cloth, or close pliable leather, six inches broad, is placed around and fastened to the edge of each of the boards, by tacks or other means; the ends having been first joined by a lap and stitched air tight seam, with not less than two rows of stitching.

The lower and upper boards are drawn together by means of an india rubber strap attached to them at the edge at which the lower hinge is. This is to keep up a constant blast, should the heel be lifted from it.

An india rubber tube, of one-fourth inch caliber, and three feet for a sitting, or four feet long for a standing bench, and armed at each end with a short, tapering metal tube, for connecting with the blow pipe, at the upper end, and inserting into the upper board of the bellows, at the lower end, serves as the conductor of the air from the bellows, on the floor, to the blow pipe in the hand.

Some small pieces of leather or wood placed under, or attached to the lower boards, to raise it one-half an inch from the floor, so as to admit air freely to the lower valve, and all is complete.

To use this bellows, place the foot across in the line of the hinges, with the toe press down the apartment opened by the wire spring; this forces the air up into the upper apartment, which is compressed in part by the india rubber strap, and partly by the heel of the foot resting on it harder or softer, as the blast is wanted stronger or weaker.

Care must be taken not to allow the tube whilst in or out of use, to bend short, as this stops the passage of air, and is very annoying to the operator. If the tube is thin, and too much disposed to bend short, it must have a coil of small wire inserted throughout its length, to keep it open.

One advantage this bellows possesses over all others, is its extreme portability. But the great one in which it excels beyond comparison is that it gives the operator complete control of the strength of the blast, enabling him to mould it at will, as effectually and with as much ease as he could with his lungs.

In a former number of the Register we alluded to this improvement; and since have been using one in our Laboratory, and find it comes up to our expectations.—[CIN. ED.

